# Prognostic value of coronary atherosclerosis and CAC score for the risk of chemotherapy-related cardiac dysfunction (CTRCD): The protocol of ANTEC study

**DOI:** 10.1371/journal.pone.0288146

**Published:** 2023-08-17

**Authors:** Anna Borowiec, Patrycja Ozdowska, Magdalena Rosinska, Agnieszka Jagiello-Gruszfeld, Slawomir Jasek, Joanna Waniewska, Beata Kotowicz, Hanna Kosela-Paterczyk, Elzbieta Lampka, Agata Makowka, Małgorzata Fuksiewicz, Magdalena Chojnacka, Agnieszka Zebrowska, Katarzyna Gepner, Aleksandra Kapala, Andrzej Cieszanowski, Zbigniew Nowecki, Jan Walewski

**Affiliations:** 1 Department of Cancer & Cardio-Oncology Diagnostics, The Maria Sklodowska-Curie National Research Institute of Oncology, Warsaw, Poland; 2 Department of Computational Oncology, The Maria Sklodowska-Curie National Research Institute of Oncology, Warsaw, Poland; 3 Department of Brest Cancer & Reconstructive Surgery, The Maria Sklodowska-Curie National Research Institute of Oncology, Warsaw, Poland; 4 Department of Radiology I, The Maria Sklodowska-Curie National Research Institute of Oncology, Warsaw, Poland; 5 Laboratory of Tumor Markers, Department of Pathology and Laboratory Diagnostics, The Maria Sklodowska-Curie National Research Institute of Oncology, Warsaw, Poland; 6 Department of Soft Tissue/Bone Sarcoma and Melanoma, The Maria Sklodowska-Curie National Research Institute of Oncology, Warsaw, Poland; 7 Department of Lymphoid Malignancies, The Maria Sklodowska-Curie National Research Institute of Oncology, Warsaw, Poland; Columbia University Medical Center, UNITED STATES

## Abstract

**Background:**

Cardiological complications of oncological treatment, including the most serious one, heart failure, constitute a significant and still unsolved clinical problem. A history of dyslipidemia and complications of atherosclerosis, including coronary artery disease, are established risk factors for cardiotoxicity in cancer patients. In recent years, a protective effect of statin treatment on the development of heart failure in cancer patients has been observed. This protocol describes a study aiming to assess the prognostic value of coronary atherosclerosis burden and the CAC score on the onset of cardiac dysfunction associated with cancer therapy.

**Methods:**

ANTEC (Atherosclerosis iN chemoTherapy-rElated Cardiotoxicity) is a single-site, prospective, observational study to evaluate the influence of the coronary atherosclerosis and CAC score assessed by computed tomography on the development of left ventricular systolic dysfunction in cancer patients with at least moderate cardiotoxicity risk. A group of 80 patients diagnosed with cancer prior to high-dose anthracycline chemotherapy (doxorubicin ≥ 240 mg / m2 body weight or epirubicin ≥ 600 mg / m2 body weight), without a history of heart failure and coronary artery disease, will be included in the study. Patient follow-up is planned for 12 months. In all patients, coronary computed tomographic angiography (CCTA) will be performed once at the beginning of the study. The primary endpoint is the onset of cancer therapy-related cardiovascular toxicity, defined as mild, moderate, severe and very severe according to ESC 2022 Cardio-oncology guidelines. During follow up, echocardiography with GLS assessment will be performed every three months. Additionally, new biomarkers of atherosclerosis (IL-6, MPO, TNF-alpha) will be measured every 6 months. The study registration identifier on clinicaltrials.gov is NCT05118178.

**Clinical trials registry:**

This study is listed on cinicaltrials.gov with identifier NCT05118178.

## 1. Background

Cancer mortality has decreased significantly over the last years due to the earlier diagnosis and increasing efficiency of chemotherapy. The benefits of administering anti-cancer drugs are to some extend limited by their adverse effects on the cardiovascular system [1–4]. The most serious cardiotoxic effect of chemotherapy is damage to the heart muscle, leading to heart failure. Cardiotoxicity is particularly important in the context of anthracycline use. The main mechanism of anthracycline cardiotoxicity is direct myocardial cell damage [5–9].

In addition, studies have shown that oncological treatment, including anthracyclines, accelerates the development of atherosclerosis and its complications [1–4]. The mechanisms by which chemotherapy with anthracyclines may causes or promote premature atherosclerosis are multifactorial. Both endothelial damage as well as arterial thrombosis, microcirculation damage and long-term changes in lipid metabolism have been reported [10–13]. However, the impact of asymptomatic atherosclerotic lesions on the occurrence of heart muscle damage in the course of chemotherapy is still an open question and currently CT angiography or CAC scoring is not routinely performed.

Risk factors for cardiotoxicity include older age, arterial hypertension, diabetes, dyslipidemia, obesity and smoking, and they coincide with atherosclerosis risk factors [1]. Several, mainly retrospective, studies with statins in the prevention of heart failure have been published, but the mechanism of their cardioprotective effect is still unknown [14–23].

Studies that define the contribution of pre-existing coronary atherosclerosis and/or its progression to toxicity during anthracycline treatment are lacking. We therefore designed our study to define both cardiac function, by echocardiogram, and coronary atherosclerosis and calcification by CCTA, at baseline and at intervals in a cohort receiving anthracycline chemotherapy.

### 1.1 Study aims

1. Assessment of the prognostic value of the coronary atherosclerosis and the CAC score before chemotherapy for the occurrence of the cardiovascular toxicity
2. Evaluation of IL-6, MPO and TNF-alpha for use in the diagnosis of cardiotoxicity during oncological treatment.
3. Influence of studied parameters, i.e. presence of atherosclerosis, echocardiographic parameters and biomarker levels on the prognosis of patients undergoing cardiotoxic chemotherapy

## 2. Methods

### 2.1 Study structure

The ANTEC Trial is a single-center, prospective, observational trial evaluating the impact of atherosclerosis in the coronary arteries determined by computed tomography on the risk of cardiotoxicity related to anthracycline-based chemotherapy. The trial is being carried out in accordance with the principles of the Declaration of Helsinki and the International Conference on Harmonization Good Clinical Practice guidelines. The conduct of the study is approved by an Independent Ethics Committee at the Maria Sklodowska-Curie National Research Institute of Oncology in Warsaw (the date of approval– 28 October 2021, approval no. 80/2021) and all participants will provide written informed consent before study entry. The registration identifier on clinicaltrials.gov is NCT05118178.

### 2.2 Study patients

The study will include patients with cancer, diagnosed and qualified for further systemic treatment with high-dose anthracycline chemotherapy at the Maria Sklodowska-Curie National Research Institute of Oncology in Warsaw. Patients must give informed and voluntary consent to participate in the study and meet all the inclusion criteria and none of the exclusion criteria listed in Table 1.

**Table 1:** Inclusion and exclusion criteria.

Inclusion criteria
1. Eastern Cooperative Oncology Group (ECOG) performance status from 0 to 2.
2. Age ≥18 years at the time of signing the informed consent.
3. Known neoplastic disease prior to the initiation of chemotherapy with a high dose of anthracyclines (doxorubicin ≥ 240 mg / m2 b.w. or epirubicin ≥ 600 mg / m2 b.w.)
4. No history of heart failure (left ventricular ejection fraction ≥ 50% as assessed by echocardiography).
5. No history of: coronary artery disease, stroke and lower limb atherosclerosis
6. At least moderate baseline cardiovascular toxicity risk according to Heart Failure Association–International Cardio-Oncology Society stratification [1]
Exclusion criteria
1. History of heart failure and/or left ventricle ejection fraction <50%
2. Significant valvular heart disease
3. Previous chemotherapy or radiation to the chest
4. Presence of any disease with a life expectancy <1 year in the opinion of the investigator.
5. Drug or alcohol abuse
6. Lack of possibility or contraindications for computed tomography (CT) scan.

### 2.3 Study visits and follow up

Approximately 80 patients are planned to be included in the study. Each patient will have 5 program visits every 3 months for 12 months. At the first visit, the patient will be informed about the type of the proposed follow-up, its course and planned tests. After signing the consent to participate in the study, patients will undergo planned procedures according to the schedule shown in Table 2.

**Table 2:** Schedule of Activities (SoA).

Visit	1	2	3	4	5
Days from first Visit window	1	90 (±14)	180 (±14)	270 (±14)	360 (±14)
Informed Consent	x				
In-/exclusion criteria	X				
Medical History/ Concomitant diagnoses	x				
Demographics	x				
NYHA classification	X	X	X	X	X
ECOG performance status	X	X	X	X	X
Physical exam	X	X	X	X	X
Concomitant Therapy	X	X	X	X	X
Assessment of Endpoints		X	X	X	X
12-lead-ECG	X	X	x	X	x
Echocardiography with GLS assessment	x	X	X	X	X
Lab test	x	X	X	X	X
CCTA	x				
IL-6, MPO, TNF-alpha	x		x		x

**Abbreviations**: ECG = electrocardiogram; ECOG = Eastern Cooperative Oncology Group; GLS = global longitudinal strain; NYHA = New York Heart Association; CCTA = Coronary Computed Tomographic Angiography

Clinical variables such as: age, gender, cardiovascular risk factors (hypertension, diabetes, hyperlipidemia, obesity, smoking status), alcohol use, previous medical history, concomitant heart failure therapy, statins or other lipid lowering medications will be documented at baseline and then checked at each visit. Lipid levels and diabetes status at each visit will be determined using a fasting blood sample and lipid panel at that visit.

Cancer diagnosis and current treatment (including cumulative dose of anthracyclines) will be collected and documented at each visit.

In all patients, a single coronary computed tomographic angiography (CCTA) will be performed at the beginning of the study, with calculation of coronary artery calcium (CAC) score according to the Agatston method. Contrast cardiac CT scanning for CAC quantification will be performed with a 64-slice CT scanner (Revolution Evo, GE Healthcare, Waukesha, USA) with prospective ECG triggering. A tube voltage of 120kVp will be used. Images will be reconstructed with a slice thickness of 0.625 mm and CACS was quantified using the Agatston method with dedicated software by a well-trained researcher.

At each visit, transthoracic echocardiography will be performed by using a Philips EPIQ Elite instrument (Philips Healthcare, Andover, Massachusetts). The following echocardiographic parameters will bez assessed: left atrium diameter, interventricular septum diameter, posterior wall thickness, left ventricle (LV) end-diastolic diameter, LV end-systolic diameter, LV ejection fraction, and mitral inflow with the use of Doppler echocardiography. Diastolic function will be evaluated by mitral inflow E/A pattern, E/A ratio, annular tissue Doppler curves and E/E’ ratio. Images will be digitally stored for offline analysis on custom software (Q-Station, Philips Healthcare, Andover, Massachusetts). Left ventricular, two-dimensional peak systolic global longitudinal strain (GLS) will be analysed by an offline semi-automated speckle tracking imaging technique from the three standard apical views. Analyses will be performed by one board-certified physician, who will be blinded to all clinical characteristics.

Blood samples will be collected et each visit and analyzed by the central laboratory (The Maria Sklodowska-Curie National Research Institute of Oncology). Laboratory tests will include: alkaline phosphatase, alanine aminotransferase (ALT), aspartate aminotransferase (AST), urea, creatinine, glucose, magnesium, potassium, sodium, uric acid, morphology, glycated hemoglobin, lipid profile, d-dimers, fibrinogen and hs-CRP.

Blood samples to determine plasma levels of high-sensitivity troponin T (hs-cTnT) and N-terminal natriuretic pro-peptide (NTproBNP) will be processed in a central laboratory using standardized methods. New atherosclerosis biomarkers (IL-6, MPO, TNF-alpha) will be assessed every 6 months using Quantikine ELISA kit R&D Systems, Inc., USA & Canada.

### 2.4 Primary and secondary endpoints

#### 2.4.1 Primary endpoint

The primary endpoint is the onset of chemotherapy-related cardiac dysfunction defined as either mild, moderate, severe or very severe according to ESC 2022 Cardio-oncology guidelines [1].

#### 2.4.2 Secondary endpoints

The secondary composite endpoint include: time to all-cause death, cardiovascular death, myocardial infarction, and stroke.

Other secondary endpoints include: (1) the severity of atherosclerosis in the coronary arteries and the calcification index in computed tomography, (2) the percentage decrease in left ventricular ejection fraction and GLS in echocardiography, (3) changes in the concentration of biomarkers involved in inflammatory and atherosclerotic processes.

## 3. Data acquisition and statistical analysis

### 3.1 Data acquisition and quality control

All measurement will be performed according to routine diagnostic standards. The critical measurements (CCTA, ECHO) will be reviewed and coded by a trained member of the study team to ensure comparability of the results. Electronic case report forms (eCRFs) will be developed to capture the study subject data in secure internal environment. The clinical data will be entered manually and laboratory data will be extracted from the Hospital Information System (HIS). The data will be pseudonimised using study subject identification number, with the list of linking these numbers to the patient identification number used in the HIS stored separately. To ensure data quality validation rules will be implemented in eCRFs to preclude entry of inconsistent or out-of-range values. Data will be assessed periodically to identify missing and outlying values to be verified with the medical notes available in HIS by the study team.

### 3.2 Sample size calculations and statistical analysis

The estimated group size was determined for the one-sided Pearson chi-square test for two proportions. We considered an alternative hypothesis of an approximately 2-fold increase in the incidence of cardiotoxicity with the presence of atherosclerotic lesions versus null hypothesis of no effect of atherosclerotic lesions. In particular, we assumed that the incidence of cardiotoxicity in the population with and without atherosclerotic lesions will vary from 60% to 75% and from 30% to 40% respectively and that the atherosclerotic lesions of various severity will be present in 30% to 40% of patients. With additional standard assumptions of 0.05 statistical significance and 80% power and assuming the estimated sample size varies from 54 to 80 individuals, thus the desired sample size of 80 was established.

Handling of missing data will depend on the proportion of cases affected by missing values. Complete case analysis will be performed if any missing values occur in less than 5% of cases. Otherwise, imputation through full conditional specification (FCS) methods are planned, accounting for the model of interest.

Statistical analysis of the results obtained in this trial will focus on the comparison of groups determined on the basis of the presence of atherosclerotic lesions, and will include a comparison of the characteristics of these groups using for comparisons of numerical variables, the Wilcoxon test, and for categorical variables the Fisher or chi-square tests. The Cochran-Armitage test on the existence of a trend for an ordinal scale variable will be used to assess the effect of the severity of atherosclerotic lesions on the incidence of cardiotoxicity.

Cumulative incidence of cancer therapy-related cardiovascular toxicity (primary endpoint) will be estimated using competing risk approach with death from other causes as the competing event. Fine and Gray’s proportional subhazards model will be used to estimate incidence by group and evaluate the impact of atherosclerotic lesions controlling for potential confounders. This model may be used in combination with missing data imputation, if necessary, with substantive model compatible fully conditional specification (SMC-FCS) imputation [24].

For time-to-event secondary endpoint (all-cause mortality) we will use the Kaplan-Meier estimates with Long-rank test to compare the survival curves. In addition, cox proportional hazard model will be used for multivariate analysis if sufficient number of events is observed. Changes in numerical variables (ejection fraction, biomarkers’ concentration) by atherosclerotic lesions status will be investigated by two-way repeated measures ANOVA.

## 4. Discussion

The influence of asymptomatic coronary atherosclerosis on the occurrence of left ventricle dysfunction in the course of oncological treatment has not been prospectively studied so far. The proposed work is the first prospective study of this type on a significant clinical problem, likely to contribute to improvement of clinical practice and the patients’ outcomes.

Atherosclerosis is an inflammatory-degenerative process which leads to cardiovascular complications. Oncological patients with cardiovascular risk factors and cardiovascular diseases are at increased risk for cancer therapy-related cardiac dysfunction [1]. Recent studies suggest cardioprotective effect of statins on cardiotoxicity during oncological treatment [14–23]. Statins lower the cholesterol level, prevent atherosclerosis development and atherosclerosis complications [13]. On this basis, the European Society of Cardiology 2022 guidelines recommend considering statins for primary prevention of cardiac dysfunction associated with cancer therapy in patients at high and very high cardiovascular toxicity risk [1]. However, there are only a few prospective studies that have shown a positive effect of statins use in cardiotoxicity prevention [14, 22]. Whether this phenomenon is related to atherosclerosis inhibition or to the pleiotropic effects of statins is still an open question. The results of our study may contribute to identification of additional patient groups, who could benefit from preventive statin treatment complementing their oncological treatment.

Cardiac computed tomography (CT) is a useful tool for measuring calcium score and atherosclerosis burden in asymptomatic individuals at risk of coronary artery disease. Calcium score improves risk classification and helps guide statin primary preventive therapy in general population [25]. In retrospective study by Brann et al., patients referred to cardio-oncology clinic had similar and less severe CAC burden compared with the general population [26]. In another study with breast cancer patients, automated CAC scoring on radiotherapy planning CT scans were performed. In 24% of patients CAC score was above zero and one in three patients with high CAC score had no other CVD risk factors [27]. A retrospective study by Hooks et al. has shown that CAC extent was a significant predictor of the coronary composite outcome, but not of the heart failure composite outcome [28]. On the other hand, in a multi-center cohort of 66,636 asymptomatic adults without cardiovascular disease (CVD), high CAC predicted the risk of death from CVD in official death certificates, independently of CVD risk factors [29]. A recent study by Kim et al., has shown that the CAC score was a significant predictor of acute coronary events in breast cancer patients [30]. However, none of these studies were designed to prospectively evaluate the impact of coronary atherosclerosis on the development of chemotherapy-related cardiac dysfunction.

### 4.1 Possible limitations

As the expected frequency of cardiotoxicity according to the new 2022 guidelines is not well established there is a risk of inappropriate assumptions for power calculations, although we adopted rather conservative assumptions. In addition, as this is an observational study confounding by other cardiotoxicity risk factors is possible. If feasible, this will be accounted for in the multivariate analysis.

## 5. Conclusion

This is the first study to prospectively evaluate the influence of asymptomatic atherosclerosis in coronary arteries on chemotherapy-related cardiac dysfunction. This work may contribute to a better understanding of the pathophysiology of cardiotoxicity and to changing the standards of management and improving survival in oncological patients.
